# Hypoxia-elicited impairment of cell wall integrity, glycosylation precursor synthesis, and growth in scaled-up high-cell density fed-batch cultures of *Saccharomyces cerevisiae*

**DOI:** 10.1186/s12934-016-0542-3

**Published:** 2016-08-15

**Authors:** Juan C. Aon, Jianxin Sun, Julie M. Leighton, Edward R. Appelbaum

**Affiliations:** Department of Microbial and Cell Culture Development, Research and Development, GlaxoSmithKline, 709 Swedeland Road, King of Prussia, PA 19406 USA

**Keywords:** *Saccharomyces cerevisiae*, Fermentation process, Scale-up, Cell wall, Exometabolomics, Metabolite profiling, Hypoxia, glycosylation pathway

## Abstract

**Background:**

In this study we examine the integrity of the cell wall during scale up of a yeast fermentation process from laboratory scale (10 L) to industrial scale (10,000 L). In a previous study we observed a clear difference in the volume fraction occupied by yeast cells as revealed by wet cell weight (WCW) measurements between these scales. That study also included metabolite analysis which suggested hypoxia during scale up. Here we hypothesize that hypoxia weakens the yeast cell wall during the scale up, leading to changes in cell permeability, and/or cell mechanical resistance, which in turn may lead to the observed difference in WCW. We tested the cell wall integrity by probing the cell wall sensitivity to Zymolyase. Also exometabolomics data showed changes in supply of precursors for the glycosylation pathway.

**Results:**

The results show a more sensitive cell wall later in the production process at industrial scale, while the sensitivity at early time points was similar at both scales. We also report exometabolomics data, in particular a link with the protein glycosylation pathway. Significantly lower levels of Man6P and progressively higher GDP-mannose indicated partially impaired incorporation of this sugar nucleotide during co- or post-translational protein glycosylation pathways at the 10,000 L compared to the 10 L scale. This impairment in glycosylation would be expected to affect cell wall integrity. Although cell viability from samples obtained at both scales were similar, cells harvested from 10 L bioreactors were able to re-initiate growth faster in fresh shake flask media than those harvested from the industrial scale.

**Conclusions:**

The results obtained help explain the WCW differences observed at both scales by hypoxia-triggered weakening of the yeast cell wall during the scale up.

**Electronic supplementary material:**

The online version of this article (doi:10.1186/s12934-016-0542-3) contains supplementary material, which is available to authorized users.

## Background

The scale-up of a *Saccharomyces cerevisiae* fermentation process from 10 to 10,000 L to produce a recombinant therapeutic protein was described previously [1]. The successful scale-up generated comparable biomass as indicated by dry cell weight (DCW), and comparable amount of product with similar quality [2, 3]. There were, however, differences in manufacturing attributes, including elevations in the weight cell weights (WCWs) and culture apparent viscosity at 10,000 L scale as compared to the 10 L scale. The oxygen transfer coefficient, *k*_L_a, and oxygen demand were shown to be key elements during the scale-up. Metabolite profiling of the extracellular culture medium (exometabolomics) revealed that several pathways were affected, including mevalonate and membrane lipid syntheses, glycolysis, the tricarboxylic acid (TCA) cycle, and the synthesis of branched chain amino acids. A common feature of these pathways is their dependence on oxygen, thus their impairment could be explained by hypoxic conditions that developed at the industrial scale. Potentially, the changes in metabolic pathways could affect cell structure and function during the fermentation process. More specifically, our previous work suggested that the metabolic changes observed at the industrial scale could affect membrane permeability, likely leading to higher cell volume (as can be judged from WCW).

There is a fundamental question of how the physiological and metabolic behaviors of a *S. cerevisiae* strain producing a recombinant protein changes in response to the transition from laboratory to industrial scale, 10,000 L. Specifically, we utilize exometabolomics to determine activation/inactivation of metabolic pathways and how they affect important physiological variables such as specific biomass and product yields but also compromising structurally the cell. Furthermore our results suggested effects due to the scale-up process on the cell wall which may have an impact on cell morphology, permeability, and resistance to mechanical forces present in highly stirred and aerated bioreactors thus explaining the differences in WCW.

The cell wall of *S. cerevisiae* represents 15 to 30 % of the dry weight, 25 to 50 % of the cell volume and is largely composed of polysaccharides and proteins [4]. Four classes of interacting components, including chitin, β 1,3 glucan, β 1,6 glucan, and mannoproteins have been reported [5]. The cell wall represents a dynamic structure that can adapt to physiological and morphological changes [6]. As a matter of fact, Aguilar-Uscanga and Francois [7] reported that hypoxia led to a 25 % reduction of the cell wall mass and to a three-fold decrease in chitin content. Yeast cells with weakened cell wall elicited by either environmental conditions or mutations, triggered a compensatory mechanism that resulted in the accumulation of mannoproteins, e.g. GPI-CWPs or Pir-CWPs, or changes in glucans, e.g. β 1,3 or β 1,6 glucan, or chitin, to avoid cell lysis 4, [6, 8–12].

Genetic, morphological, and biochemical evidence shows a critical link between N- and O-types of glycosylation with the assembly and integrity of the cell wall in *S. cerevisiae* [12, 13]. Impairment of N-glycosylation led to β 1,6 glucan loss and a more diffused outer layer of the cell wall [12]. On the other hand, Willer et al. [14] showed that lack of O-mannosylation can cause abnormal cell wall and septum formation. Our previous findings already showed higher levels of ergosterol precursors like 3-hydroxy-3-methylglutarate and acetoacetate, and membrane components like choline, glycerol 3-phosphate, and glycerophosphorylcholine, at 10,000 L scale than at 10 L scale, without changes in cell viability. At industrial scale results indicated a defective synthesis of sphingolipids and ergosterol [1]. Then, it is known that a defective synthesis of sphingolipids and ergosterol impairs the incorporation of Gas1p (a GPI-anchored α-1, 3-glucanosyltransglycosylate) to the cell wall [15], and consequently the reduced Gas1p incorporation increased cell wall porosity due to reduced β-glucan crosslinking [16].

Under the hypothesis that the weakening of the yeast cell wall arises as a result of conditions imposed by the scaling up process, in this study we combine exometabolomics with analysis of cell wall integrity to further understand the mechanisms underlying this phenomenon.

## Results

### Cell growth based on volume fraction occupied by cells and specific growth rates at two scales

There was a clear difference in the volume fraction occupied by cells as revealed by WCW measurements of yeast cultured under laboratory (10 L) versus industrial (10,000 L) scales after an elapsed fermentation time (EFT) 60 h. Hypothetically, the observed increase of WCWs may be explained by an increase in cell size. Evidence showing cell cycle-related size change versus growth rate in *S. cerevisiae* carbon-limited chemostat cultures was reported previously [17, 18].

**Fig. 1:**
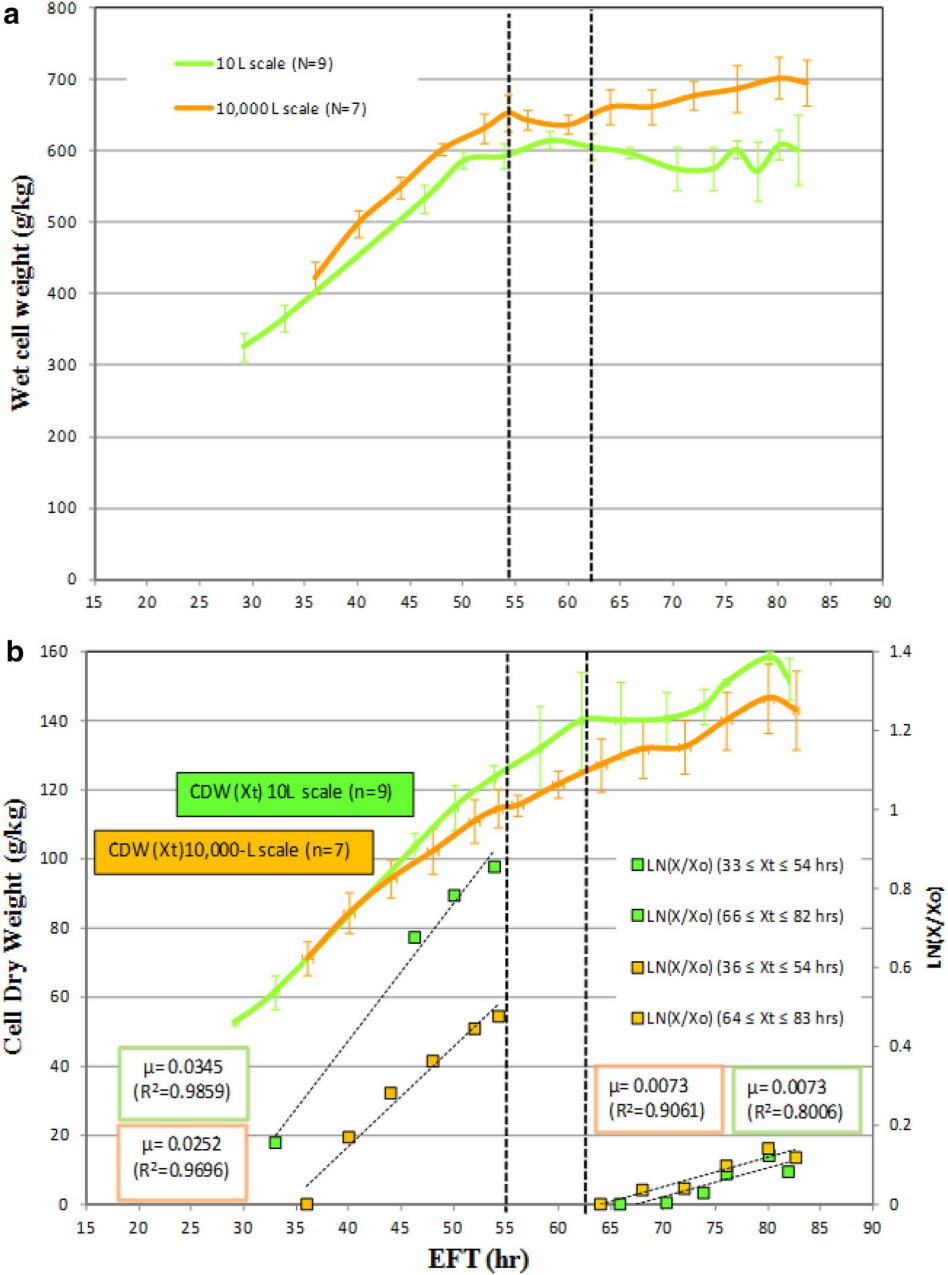
Comparison of wet cell weight (**a**) and cell dry weight (**b**) at 10,000 and 10 L scale. The *green* and *orange* lines represent the temporal biomass accumulation profiles at 10 and 10,000 L, respectively. In **b** the specific growth rates are from the best linear fit of the semi-LN plot of CDW versus elapsed fermentation time (EFT) in hours (hr). Plotted CDW, WCW values are average of 9 and 7 batches at 10 and 10,000 L scale, respectively. In both panels the two parallel-*dashed lines* delimitate the transition time between fast- and slow-growing cells during fed-batch. *Error bars* represented one standard deviation

The results depicted in Fig. 1a show WCWs determined at 10,000 L scale trending similarly but always slightly higher than those at 10 L scale between EFT 33 and 55 h. Fig. 1b shows cells at 10 L scale growing at higher specific growth rates (µ) per CDW than those at 10,000 L scale. In Fig. 1a, shows that after EFT 83 h the difference between WCWs at the two scales was magnified whereas µ was not significantly different (Fig. 1b). The two vertical black dashed lines in panel b indicate the transition phase corresponding to the accumulation of biomass. Before EFT 54 h, WCW accumulation increased fast at both scales; however, after EFT 64 h, WCW increased slowly at 10,000 L while leveling off at 10 L. WCW at 10,000 L was trending higher than at 10 L during the whole fermentation process. Before EFT 54 h, the rates of CDW accumulation at 10 L and 10,000 L were 0.0345 and 0.0252 h^−1^, respectively whereas after EFT 64 h the µ decreased dramatically to 0.0073 h^−1^ at both scales. Cells at 10,000 L could be under challenging conditions as reflected by similar CDWs than those measured at 10 L scale between EFT 60 and 83 h, but higher cell volume measured as WCW compared to 10 L as shown by panels a and b in Fig. 1.

In principle, the ensemble of data indicates that a positive correlation between growth rate based on CDW and cell’s volume cannot explain the results. We suspect the aggravating effect of hypoxia not only upon anabolism/catabolism but also on the structure/functionality of membranes and other important cellular components that play a key role in maintaining the yeast cell wall integrity thus mediating cell volume changes.

### Cell wall integrity by zymolyase assay

To test whether metabolic changes triggered by hypoxic conditions existing at the industrial scale affected cell wall integrity, we implemented a Zymolyase assay that probes the cell wall sensitivity to the enzyme’s activity as detailed in “Methods” section.

**Fig. 2:**
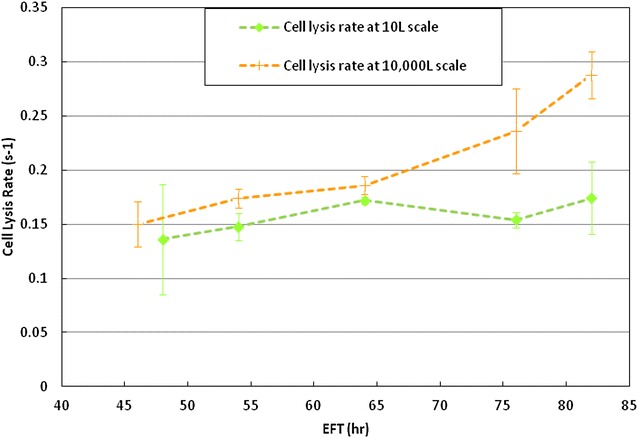
Comparison of cell lysis rates (s^−1^) shown by the PRD strain throughout the fermentation time between scales 10 and 10,000 L scale. The *green* and *orange dashed lines* represent the cell lysis rates profiles at 10 and 10,000 L, respectively. Those cell lysis rates were calculated as described in “Methods” section. All rate determinations at 10 L were by duplicate. All rate determinations at 10,000 L were by triplicate.* Error bars* represented one standard deviation

First, we comparatively examined the cell wall integrity of the production strain (PRD) at both scales. Samples were collected at EFT 46, 54, 64, 76 and 80 h, re-suspended in PBS, and tested for Zymolyase sensitivity. Figure 2 shows the results obtained with the PRD strain at 10 and 10,000 L. At 10 L cell lysis rates increased from 0.14 to 0.17 s^−1^ or a 21 % rise throughout the course of the fermentation. In contrast, at 10,000 L the PRD strain showed a rising trend of cell lysis rates starting at 0.15 s^−1^ (EFT 46 h) and up to 0.29 s^−1^ thus more than 50 % increase after EFT 64 h. The cells from EFT 76 and 80 h were significantly more sensitive to Zymolyase activity at 10,000 L than at 10 L scale.

The results obtained indicate that the PRD cell wall exhibits a progressive weakening as the fermentation process progresses at the industrial scale. These results suggest that the cell volume increments seen at 10,000 L scale could be caused by the cell wall weakness. Then our hypothesis that the hypoxia compromised the cell wall biogenesis and its integrity of those cells cultured at large scale needed to be investigated requiring more data from exometabolomics.

### Other potential microorganism- or bioprocess-dependent factors influencing cell wall sensitivity

#### Heterologous protein expression

We tested whether the metabolic burden introduced by the high expression of heterologous protein Pr-1 by affecting cell proliferation and cell growth, e.g. by competing for key metabolic intermediates, interferes with cell wall biogenesis.

**Fig. 3:**
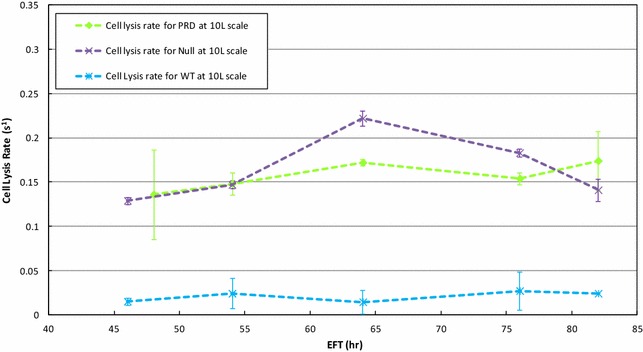
Cell lysis rate comparisons between the production (PRD) strain, the non-producing null strain, and the wild type (WT) strain at 10 L scale. The *green*, *purple*, and *blue dashed lines* represent the cell lysis rate profiles of PRD, null, and WT, respectively, at 10 L. Those cell lysis rates were calculated as described in “Methods” section. All rate determinations were by duplicate. *Error bars* represented one standard deviation

The PRD and Null strains were run under optimized production conditions at 10 L to investigate the cell wall sensitivity to Zymolyase (Fig. 3). Genotypically, the only difference between the PRD and Null strain was the presence or absence, respectively, of the Pr-1 coding region for the heterologous protein. The Null strain exhibited cell lysis rates ranging from 0.13 to 0.22 s^−1^ up to EFT 64 h; and decreasing to 0.14 s^−1^ thereafter. Compared to the cell lysis rates shown by the PRD strain (from 0.14 to 0.17 s^−1^, Fig. 2), there were no significant differences except for EFT 64 h, and the reason for this unexpected high cell lysis rate is unclear. However, both strains exhibited minimum changes in the sensitivity to Zymolyase over time at the 10 L scale, indicating the lack of contribution by the therapeutic protein expression to the weakening of the cell wall.

In addition, an ATCC strain named a null-wild type (WT) strain was included and used to run the same process at 10 L to compare the level of sensitivity to Zymolyase with our production Null strain. Figure 3 showed the significant lower cell lysis rates of the WT strain when compared to the Null and PRD strains during the entire fermentation time at the same 10 L scale. Clearly these lysis rates are providing references of cell wall integrity relatively to other host not related to the PRD one. Then, this PRD host strain exhibited inherently lower cell wall integrity but hypoxia at the 10,000 L, progressively aggravated it throughout the fermentation time.

#### Biomass yield and cell viability

As shown in Table 1, the partial volume occupied by cells, as reflected by WCW, was consistently 16 % higher at 10,000 L than at 10 L (Fig. 1) by comparing PRD at both scales. This result is in agreement with a higher WCW/CDW ratio for the PRD strain at 10,000 L (Table 1). However, viability and biomass yield, did not exhibit significant differences with respect to the scale of production.

**Table 1:** Average viability and biomass comparison between the production (PRD), the Null and wild-type (WT) strains

	PRD 10,000 L, n = 4	PRD 10 L, n = 9	Null 10 L, n = 2	WT 10 L, n = 2
Viability (%)	93.1	93.0	93.6	98.0
CDW (g/kg)	143	152	157	210
WCW (g/kg)	696	602	636	714
WCW/CDW	4.87	3.96	4.05	3.40
Biomass yield (g/mol glucose)	70.2	70.2	70.8	93.4

At 10 L scale, the WT strain showed higher CDW and WCW of 210 and 714 g/kg than 157 and 636 g/kg for Null production strain. These results are also reflecting in better biomass yields on glucose by WT host strain, 93 g/mol, than those measured in the PRD-related host strains of slightly above 70 g/mol (Table 1). Using the ratio of WCW to CDW the WT strain exhibited the lowest ratio compared to the production host, indicating the least volume occupied by the cell per unit of biomass measured as CDW.

These results are indicating the nature of metabolic changes related to the cell wall biogenesis and its integrity as suggested previously.

#### Production process stage

**Fig. 4:**
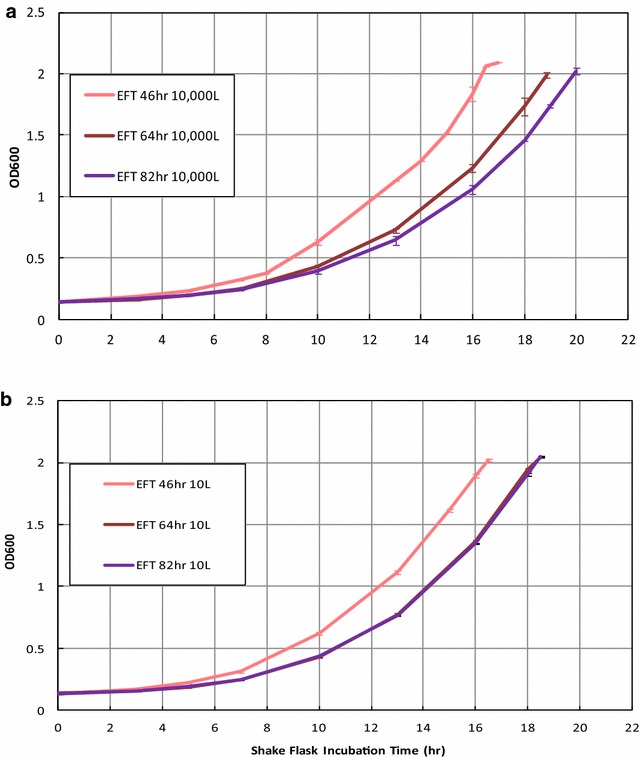
Growth curves for inoculums from 10,000 and 10 L sampled at EFT 46, 64, and 82 h. **a** Optical densities at 600 nm (OD_600_) versus incubation time of corresponding shake flasks inoculated with samples at EFT 46 h (*pink*), 64 h (*brown*), and 82 h (*purple*) from 10,000 L production bioreactors. **b** OD_600_ temporal profiles corresponding to shake flasks inoculated with samples at the indicated EFTs from 10 L production bioreactors. Optical densities at 600 nm (OD_600_) measured as detailed in “Methods” section. All determinations are by duplicate. *Error bars* represented one standard deviation

We investigated the capacity of yeast cells to reinitiate the mitotic cycle as a function of the bioprocess stage to, ultimately, understand the metabolic bases of the PRD differences between scales. We analyzed the growth kinetics when yeast cell were re-grown in shake flask containing fresh medium. Cells were withdrawn from the production bioreactor at EFT 46, 64 and 82 h at both scales, resuspended in fresh medium and their growth kinetics evaluated based on µ, the shake flask incubation time to reach a target OD_600_ of 2 (mid-exponential phase, Fig. 4) as well as the total glucose consumed and ethanol produced and their corresponding rates (Table 2). At 10,000 L scale, cells from EFT 46 h exhibited the highest specific growth rate of 0.193 h^−1^ whereas cells harvested later progressively lowered the specific growth rates from 0.171 h^−1^ at 64 h to 0.164 h^−1^ at 82 h. Interestingly, cells from EFT 64 or 82 h showed longer lag phases than at EFT 46 h (19 and 20 h versus 16.75 h, Fig. 4a). Consistently with the typical glucose over-flow metabolism known in *S. cerevisiae*; cells harvested at 46 h consumed glucose and generated ethanol faster as compared to 64 and 82 h (Table 2). Overall, the results show that cells harvested later in time were kinetically slower to re-initiate the mitotic cycle, exhibiting approximately a delay of 2 to 3 h.

**Table 2:** Summary of growth kinetic, glucose consumption, and ethanol production of samples from 10,000 and 10 L scales

	10,000 L			10 L		
	46 h	64 h	82 h	46 h	64 h	82 h
Time to reach OD = 2 (h)	16.8	18.9	20.0	16.5	18.5	18.5
Glucose consumed (g/L)	12.1	12.6	12.2	12.3	13.0	12.7
Glucose consumption rate (g/L/h)	0.722	0.664	0.608	0.745	0.700	0.686
Ethanol produced (g/L)	4.37	4.32	4.38	4.54	5.05	4.72
Ethanol production rate (g/L/h)	0.261	0.229	0.219	0.275	0.273	0.255
Specific growth rate (h^−1^)	0.193	0.171	0.164	0.193	0.184	0.181

A similar study was performed in the 10 L bioreactors. As shown in Fig. 4b, cells harvested earlier grew faster and exhibited shorter lag phases than those harvested later. Those cells harvested at later EFT 64 and 82 h exhibited the µ of 0.184 and 0.181 h^−1^ as shown in Table 2. Those cells from EFT 64 or 82 h showed longer (18.5 h) lag phases than those harvested at EFT 46 h (16.5 h) to reach same OD_600_ of 2. Following the typical glucose over-flow metabolism of *S. cerevisiae*; ethanol production rates were higher at EFT 46 h compared to those rates measured in late time at 10 L scale (Table 2).

When comparing between scales, however, cells harvested at early time points (EFT 46 and 64 h), similar growth rates, lag phase lengths, and total glucose consumed can be found but still glucose uptake and ethanol production rates are higher at 10 versus 10,000 L indicating different enzyme make-ups required for glycolysis probably induced by the previous hypoxic conditions in the production bioreactor (Table 2). Additionally cells from 10 L scale were faster to re-initiate the vegetative growth in shake flasks than those from 10,000 L scale, especially when withdrawn later from the production bioreactor (Fig. 4).

### Hypoxia-elicited impairment of glycosylation pathway precursors and its impact on cell wall biogenesis and integrity as revealed by increased leakiness

Our previous work suggested that hypoxia triggered down-regulation of the mevalonate pathway at the industrial scale as compared to the laboratory scale [1]. In yeast, the mevalonate pathway is important not only for the biosynthesis of ergosterol, the main sterol presents in cell membranes, but also for generating dolichol phosphate (DolP), a lipid acceptor of sugar residues, particularly mannose to form DolP mannose (DolP Man). This sugar-lipid intermediate is involved in the N- and O-glycosylation at the golgi, including the synthesis of the sugar part of the glycosyl phosphatidyl inositol (PGI) anchor as well as of proteins with internal repeats (Pir-CWP) [5].

**Fig. 5:**
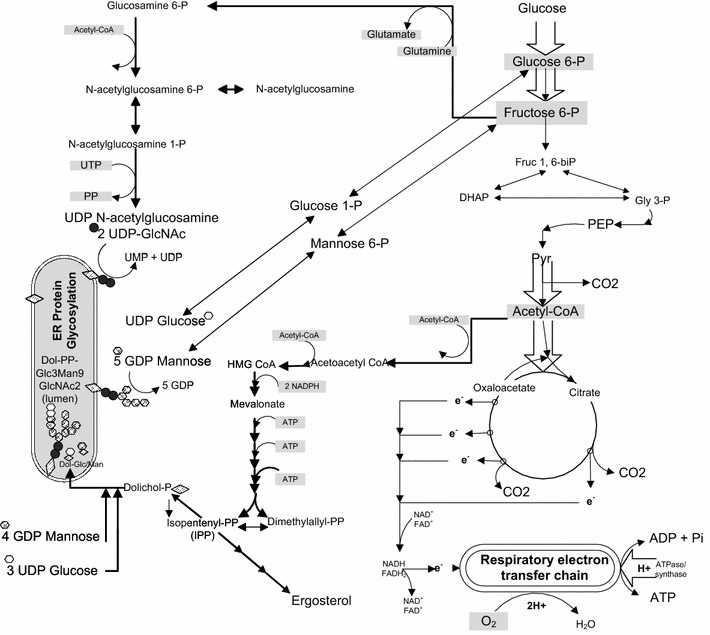
N-glycosylation linkages are derived from *N*-acetyl-glucosamine (GlcNAc), mannose (Man) and glucose (Glu). These sugars are added consecutively onto dolichol, a poly isoprenoid lipid carrier embedded in the endoplasmic reticulum (ER) membrane. 3 UDP Glu and 4 GDP Man from the cytoplasm were bound to dolichol first via a pyrophosphate linkage (–PP–). After the Man5GlcNAc2-PP-dolichol intermediate is completed, the entire complex is flipped into the lumen of the ER

**Fig. 6:**
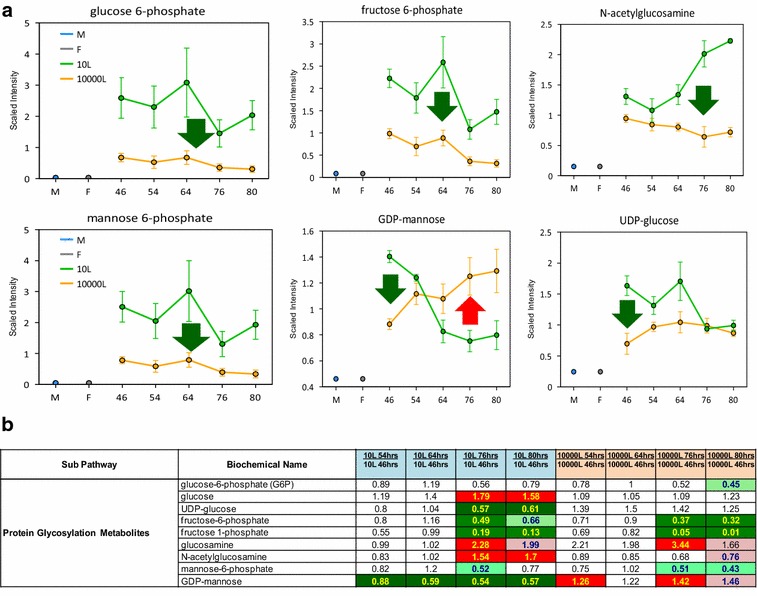
Line plot graphs from 10 L (*green line*) 10,000 L (*orange line*) scale and the corresponding heat map table showing metabolites related to protein glycosylation pathways. **a** *Line plots* graphs of the glycosylation pathway related intermediates comparison between scales. **b** Heat map table. The heat map tables are color coded, and descriptions are shown in Additional file 1: Figure S1. The intermediates measurement and data analysis were determined as described in “Methods” section. *Error bars* represents “mean ± one standard deviation”. “M” and “F” in the time axis for every line plot stand for medium and feed samples, respectively

Several metabolic pathways are known to contribute to protein glycosylation, and intermediates of these pathways were scrutinized for differences over time and between the two scales (Fig. 5). Glucose-6-phosphate (G6P), fructose-6-phosphate (F6P), mannose-6-phosphate (Man6P), UDP-glucose (UDP Glu) and N-acetyl-glucosamine (GlcNAc) were all consistently at lower levels in the 10,000 L compared to 10 L reactor thus suggesting differences in protein glycosylation. More specifically, GDP-mannose (GDP-Man), an important upstream sugar lipid intermediate for O- and N-glycosylation, increased over time in 10,000 L whereas it decreased in the 10 L system (Fig. 6). The lower levels of Man6P and the progressively higher amounts of GDP-Man detected suggest partial impairment of protein glycosylation from GDP-Man at co- or post-translational glycosylation levels in the 10,000 versus 10 L that aggravated as a function of time.

**Fig. 7:**
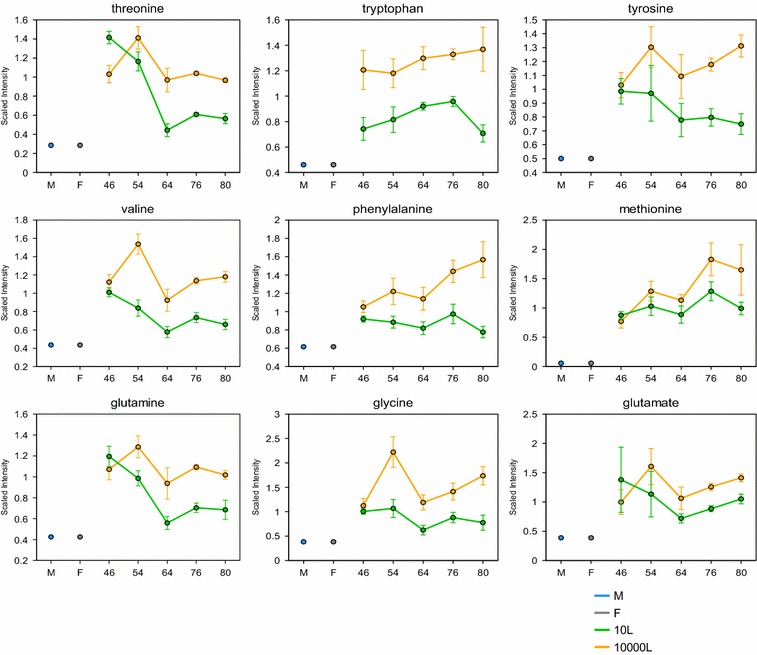
Line plot graphs of amino acids in the media from 10 L (*green line*) and 10,000 L (*orange line*) fermentations. The intermediates measurement and data analysis were determined as described in “Methods” section. *Error bars* represents “mean ± one standard deviation”. “M” and “F” in the time axis for every line plot stand for medium and feed samples, respectively

**Fig. 8:**
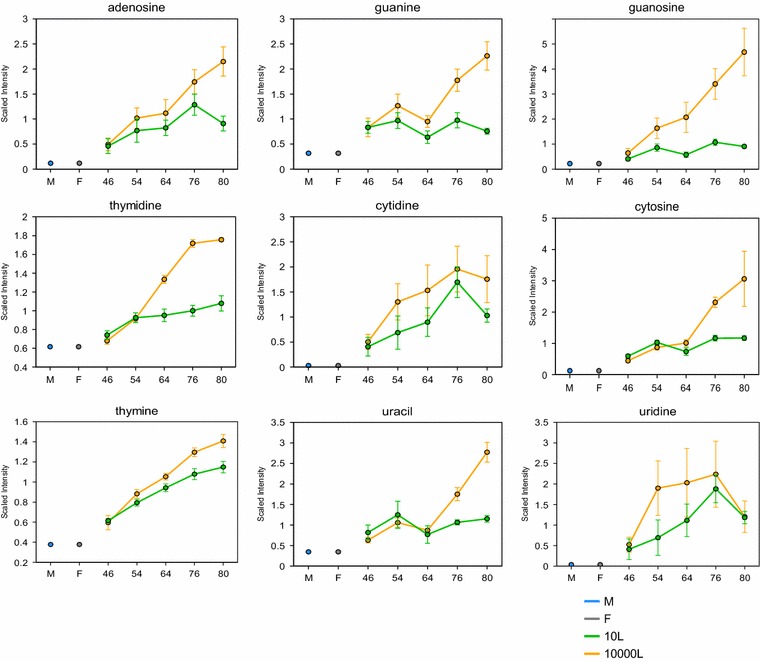
*Line plot graphs* of several nucleic acids in the media from 10 L (*green line*) and 10,000 L (*orange line*) fermentations. The intermediates measurement and data analysis were determined as described in “Methods” section.* Error bars* represents “mean ± one standard deviation”. “M” and “F” in the time axis for every line plot stand for medium and feed samples, respectively

Taking into consideration these results suggesting a cell wall integrity highly compromised, now further supported by more exometabolomics data showing the leak of key metabolites for biomass synthesis/maintenance (Figs. 7, 8). The leakiness of the yeast cell walls was reflected by the appearance in the extracellular medium at 10,000 L scale of higher levels of several amino acids, e.g. threonine, phenylalanine, glutamine, methionine, tyrosine, tryptophan, valine, and glycine than those analyzed at 10 L (Fig. 7). At 10,000 L scale, there was also noticeable accumulation of nucleic acids, purines and pyrimidines, e.g. thymidine, adenosine, guanine, guanosine, cytosine, and uracil compared to 10 L scale (Fig. 8).

The data presented show that the glycosylation pathway in yeast offers many levels of possible regulation which might influence the cell wall integrity and so leakiness of metabolites. Likely, these effects may be mediated by a differential utilization of metabolic precursors of protein glycosylation occurring at the two scales.

## Discussion

The main contribution of this work is a detailed characterization of the effects triggered by hypoxia on the physiological behavior of *S. cerevisiae* during the scale up of a high-cell density fed-batch process for the commercial production of a therapeutic protein. Herein, using exometabolomics, we further show that impairment of glycosylation pathway triggered by hypoxia at 10,000 L affect the cell wall integrity of *S. cerevisiae*, likely due to low supply of phosphorylated sugars namely GDP mannose.

Previously, we reported on the process control improvement to mitigate the scaling up effect on performance as measured by productivity and product quality. However, irrespective of those changes, the WCW and broth viscosity differences between laboratory and industrial scales remained. Exometabolomics data further revealed that those physiological differences could be explained by low availability of dissolved oxygen. Apparently, the impact of hypoxia was not only on the functionality of metabolic pathways like (i) mevalonate/ergosterol, (ii) glycolysis/TCA cycle [1], but also included cell structures like (iii) the cell wall and its physico-chemical properties. Zymolyase activity exposed a defect in the cell wall integrity at the industrial scale. Exometabolomics confirmed impaired function of glycosylation pathways that supply intermediates for cell wall biogenesis while indicating leak of several key building blocks for biomass, amino acids, nucleotides and nucleosides.

The mevalonate pathway is known to affect post-translational modification of proteins such as protein glycosylation [16, 19]. Protein glycosylation requires DolP, which is synthesized together with other isoprenoid lipids in the mevalonate pathway. Protein glycosylation associated with the secretory pathway in *S. cerevisiae* involve attachment of N-linked saccharides to asparagine, O-linked mannose glycans to serine or threonine residues, and anchoring of glycosyl phosphatidyl inositol (GPI) to the –COOH terminus of membrane proteins [20].

*N*-glycosylation of protein utilizes DolP as a lipid acceptor of sugar residues. Within the ER, DolPP-GlcNAc2Man5 is converted to DolPP-GlcNAc2Man9Glu3, using DolP Man and dolichol phosphate glucose (DolP Glu) as sugar donors [21]. Also DolP Man is a substrate for protein O-mannosylation, where it serves as the donor of the first mannose to be attached to hydroxyl groups of serine and threonine in the protein. The second and subsequent mannose residues are transferred directly from GDP Man [22]. These glycoproteins are secreted into the periplasmic space and are incorporated into the rigid cell wall structure as mannans [20]. In addition DolP Man is involved in the synthesis of the sugar moiety of the GPI anchor in yeast and other eukaryotes [20]. Therefore, a link between the cell wall integrity and the levels of phosphorylated sugars (e.g. GlcNAc 6P, glucose 6P, glucose 1P, mannose 6P), sugar nucleotides (e.g. UDP-GlcNAc, GDP Man, UDP Glu), and DolP is possible. Our exometabolomics data showed low levels of phosphorylated sugars, e.g. G6P, fructose 6P, and mannose 6P as well as GlcNAc (surrogate of GlcNAc 6P) at 10,000 L compared to 10 L scale (Fig. 6). Furthermore, the accumulation of nucleotide sugars GDP Man and UDP Glu pointed out a possible decrease in their utilization by the posttranslational glycosylation pathway, at late stages of the process in both scales analyzed.

**Fig. 9:**
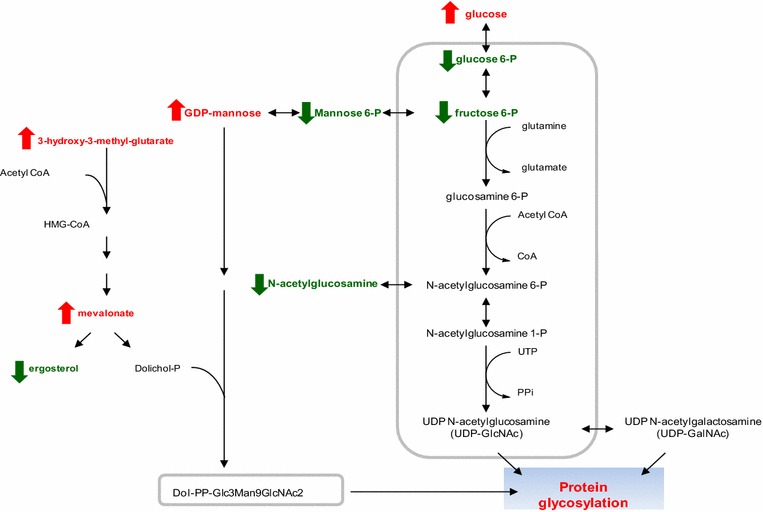
Pathway diagram of precursor synthesis for protein glycosylation and mevalonate pathway. Results show increases in upstream intermediates (*red arrows*) and significant decreases in proximal intermediates (*green arrows*) between the two scales, 10 and 10,000 L

A pathway diagram of metabolic pathways contributing to protein glycosylation showing significant increases in upstream intermediates (red arrows) and significant decreases in proximal intermediates (green arrows), which suggest potential differences the glycosylation pathway between the two bioreactor scales (Fig. 9). Accumulation of mevalonate and GDP-mannose indicates lack of glycosylation intermediates utilization, suggesting partial inhibition of glycosylation pathway in cells cultured at 10,000 L scale with respect to those at 10 L scale.

The connection of the cell wall integrity and glycosylation pathway has been reported based on genetic, morphological, and biochemical evidence in *S. cerevisiae* [12, 13]. It was reported that strains with a significant loss of the outer mannoprotein layer as compared with the wild-type exhibit a diffused cell wall [12]. Furthermore the resistance of yeast cells to Zymolyase as an indicator of cell wall integrity was reported increased with the increased levels of β-1,6-glucan [23]. In this work we reported the cell wall integrity based on the cell wall sensitivity to Zymolyase activity measured with cells from different times points and at 10 and 10,000 L scales. We considered these measurements based on cell lysis rates as reflection of the functional/structural status of the yeast cell wall. At 10,000 L compared to 10 L, particularly at late time points, the production strain showed a significant increment in the cell lysis rate (Fig. 2). But at 10 L scale, the production (PRD) strain and the Null strain did not show clear increasing trends of the sensitivity to Zymolyase activity throughout the fermentation time. Any possible contribution by the over-expression of the therapeutic protein to the compromise of the cell wall integrity was not found (Fig. 3). Then the metabolic burden represented by the significant expression of the heterologous protein of interest mostly affected yeast cells by slowing down growth after EFT 64 h but not compromising the cell wall integrity.

Taking into consideration the significant difference of the cell lysis rate at late time points between scales proved the hypothesis about the cell wall integrity being affected by hypoxia. Comparatively to other host strains, an ATCC strain named a null-wild type strain was included and found out the significant lower cell lysis rates when compared to the Null and PRD strains during the entire fermentation time at the same 10 L scale. Then the wild type strain exhibited the lowest WCW/CDW ratio compared to the production host, indicating the least volume occupied by the cell per unit of biomass. This result, combined with a higher resistance to Zymolyase activity demonstrated the correlation between cell wall integrity and the relative volume occupied by cells.

The data reported in this study proved that hypoxia aggravated the cell wall integrity when the process was scaled-up to 10,000 L and even more clear seen at the late time points. Furthermore, metabolomics data supported the idea of a compromised cell wall integrity as reflected in the leakiness of key biomass intermediates in late time points at 10,000 L higher extracellular levels of several amino acids and nucleic acids than those at 10 L scale (Figs. 7, 8). Then those cell volume increments seen at 10,000 L scale could be explained by the cell wall weakness and so compromising the four major functions: (i) stabilization of internal osmotic conditions resulting in limiting water influx, (ii) protection against physical stress, or external hydrostatic pressure (iii) scaffold to external layer of highly mannosylated proteins, and (iv) maintenance of cell shape [24]. Considering the situation of yeast’s weaken cell wall, the high hydrostatic pressure and sheer stress present at the industrial scale could contributed to cell volume changes and significant leakiness of key intracellular metabolites, in addition to the hypoxic conditions. Further investigation is required to document morphological and biochemical changes during the yeast life cycle at both fermentation scales.

We examine the impact of hypoxia on the reversibility and magnitude of the cell structural/functional impact. When cells were removed from different fermentation scales and re-initiated growth in fresh media, the reversibility of the functional/structural impact by hypoxia was revealed. There was also positive correlation between the length of the cell exposure to hypoxia under the industrial process conditions and the time to re-initiate the vegetative growth in shake flasks (Fig. 4). Underlying this correlation, there is possibly a fundamental connection between the specific growth rate, the relative volume occupied by the cells, and the magnitude of the structural/functional cell impairment. Aon and Cortassa described a correlation between the growth rate and length of G0/G1 phase of *S. cerevisiae* life cycle [17]. Longer cells lagging in G0/G1 phase correlated with lower specific growth rates. Additionally flow cytometry and fluorescence microscopy results showed either low percentages of budded cells or those with a single content of DNA per cell when a wild type strain was grown at low dilution rates in chemostat cultures [17]. These findings plus the results in this study could be explained by a connection between the specific growth rate, the WCW, and the magnitude of the structural/functional cell impairment. As shown in our Fig. 4; Table 2 at both scales the specific growth rates were lower at later, from EFTs 64 and 80 h, than early time points suggesting the possible longer residence of yeast cells in G0/G1 phase with a consequent growth in cell volume as reflected by the measurements of volume fractions occupied by cells at both scales [17, 18]. But the difference at 10,000 L scale where those WCWs were larger than those measured at 10 L indicate the contribution can be explained by the impairment of the metabolic pathway functionalities and cell wall integrity induced by the hypoxia phenomenon. Furthermore, we could discern specifically by combining the contributions of both: (i) the 10,000 L industrial scale and (ii) the very late time point, 80 h versus 64 h, the higher cell lysis rates as reflection cell wall integrity impacted on a longer lag phase or time to re-initiate growth in shake flasks (Fig. 4a). At 10 L cells harvested at both time points, 64 and 82 h, exhibited no difference in the time to reach OD_600_ 2 and the specific growth rates (Fig. 4b; Table 2), but at 10,000 L, there were differences in both same variables indicating the presence cells with severely compromised cell wall and unbalanced cell energy metabolism.

The inclusion of the temporal profiles of glucose consumption and ethanol production augmented the idea when levels of oxygen and fresh media components are re-established, the yeast cells with weaker cell walls harvested from late fermentation time point 80 h, took longer to re-establish the intracellular milieu favorable to pass G0/G1 phase and re-start the vegetative growth. Another point to include in this discussion, as reported here and in our previous publication [1], at 10,000 L scale cells exposed to a more severe hypoxia supported by the increasing impairment of oxidative phosphorylation possibly induced the arrest of vegetative (proliferation) cell growth, having a larger population increasing their cell volume.

## Conclusions

We have reported how this environmental condition, hypoxia, generated at industrial scale, induced physiological changes explaining the differences in the manufacturing attribute wet cell weight. Exometabolomics and cell wall integrity based on sensitivity to Zymolyase gave us an important insight to understand the metabolic pathways impairments as their connection to the compromised cell wall integrity. Both sets of results supported (i) the observed delays to re-establishing vegetative growth under favorable conditions and (ii) the greater cell leakiness in the late time points in this particular fermentation process at industrial scale.

## Methods

### Strains

The *S. cerevisiae* production strain (PRD) was originally developed from the parental *S. cerevisiae* strain AH22 (ATCC 38626), with the gene encoding the product (Pr-1) in a high-copy plasmid [1]. Host strain was expressing and secreting the Pr-1 molecule under a glucose limiting condition. Null strain in this study refers to the same strain but with the plasmid lacking the encoding sequence for Pr-1. Wild type strain used in this study was ATCC YHS74 (MATa *cir leu2*-*3,*-*112 pep4*-*3 prb1*-*1122 prc1*-*407*), and YHS74 was engineered on ura3-52 mutation reversion from ATCC’s AB122 strain.

### 10 L fermentation process

The details of the 10 and 10,000 L scale have been reported before [1]. Briefly, the seed train started in a shake flask incubated and agitated in a rotary shaker at 30 °C and 225 rpm until glucose was depleted. Then the flask culture was used to inoculate a seed bioreactor (Biolafitte, Saint Germain en Laye, France) to achieve higher cell density with the following set points: backpressure 10 psi, dissolve oxygen (DO) 30 %, pH 6.0 and temperature 29 °C. This seed stage consisted of a batch phase followed by a fed-batch phase using glucose nutrient feed. The pH and DO control loops were the same as described before via Distributed Control System (DCS, Siemens Moore APACS, US) [1]. DO was controlled at a cascade system of automatically first ramping up agitation until the maximum (900 rpm), and then injecting pure oxygen being blend into the inflowing air fixed at a total gas sparge rate if needed. The pH was controlled using ammonium hydroxide (30 %) and phosphoric acid (17 %) additions. Once the target optical density (OD_600_) of 100 ± 20 was reached, the culture was used to inoculate a 10 L production bioreactor with a target inoculation of OD_600_ 15. The production process was a similar process to the seed expansion with backpressure 10 psi, DO 12.5 %, pH 5.75 and temperature 29 °C. A glucose nutrient feed and a phosphate salt solution feed were both initiated when glucose concentration was ≤0.3 g/L in the batch medium. The shake flask medium [modified buffered minimal medium (BMM)], seed media (modified MW10 medium) and feed media (modified MW10 medium) were the same as described in Fu et al. [1]. Samples were taken at certain intervals to monitor biomass, as well as metabolites such as glucose and ethanol. Glucose and ethanol concentration was measured offline with a YSI biochemistry analyzer (YSI, OH, US). OD_600_, WCW, DCW and so on were tested as in the analytical method section. The fermentation process was terminated after 80 h of fed-batch time. At pre-determined time points, fermentation supernatants and pellet samples were collected for metabolite profiling and Zymolyase assay, respectively.

### 10,000 L fermentation process

A full seed train was implemented, including two stages of shake flask cultures and two stages of seed bioreactors. The scale-dependent process parameters such as batch volume, glucose nutrient and phosphate salt feed rates as well as total gas flow rate were accordingly up-scaled as detailed in Fu et al. [1]. At pre-determined time points, fermentation supernatants and pellet samples were collected for metabolite profiling and Zymolyase assay, respectively.

### Wet cell weight and cell dry weight determination

At periodic intervals, samples were taken for determination of wet cell weight (WCW) and cell dry weight (CDW). The details of WCW and CDW determinations are detailed in Fu et al. [1].

Accumulation of CDW (µ) was also calculated in this study, following

$$\upmu = \ln \left( {{\text{CDW}}_{2} / {\text{CDW}}_{1} } \right)/({\text{t}}_{2} - {\text{t}}_{1} )$$

where t_1_, t_2_ represent the time of sampling and CDW_1,_ CDW_2_ are the CDWs taken at t_1_, t_2_

### Growth kinetics in shake flask exhibited by cells from 10 to 10,000 L bioreactors

Samples from 10 L or 10,000 L bioreactors were taken at pre-determined elapsed fermentation times (EFT), 46, 64 and 82 h, were diluted to OD 45.15 using fresh shake flask medium, and then used as inocula for studying growth. 1 mL inoculum was added to 300 mL of shake flask medium (BMM) contained in a 1 L shake flask. Samples were taken from the shake flask aseptically every 2 to 3 h as needed until OD 2 was reached. OD, glucose and ethanol concentrations were measured for the samples from shake flasks. The experiments were performed in duplicate, and all values reported are averages.

Specific growth rate (µ) was calculated according to

$$\upmu = \ln \left( {{\text{OD}}_{2} /{\text{OD}}_{1} } \right)/({\text{t}}_{2} - {\text{t}}_{1} )$$

where t_1_, t_2_ represent the time of sampling, OD_1_ and OD_2_ are the ODs taken at t_1_ and t_2_

### Zymolyase assay for cell wall integrity

Zymolyase assay was used to obtain the cell lysis rate, which indicates the weakness of yeast cell wall. Then the cell wall integrity was evaluated by Zymolyase defined as a cocktail of 1, 3-β-glucanase and protease activities commonly used to degrade yeast cell walls [25, 26]. The protocol was modified from Aguilar-Uscanga and Francois [13]. The higher the cell lysis rate the weaker the cell wall. Samples were tested for Zymolyase sensitivity by diluting fresh culture sample in 10 mM Tris–HCl (pH 7) to a final optical density at 600 nm (OD_600_) of approximately 0.4. The Tris buffer contained 0.25 µg of Zymolyase 100T (MP Biomedicals) per ml. Subsequently, the decrease of the OD_600_ was monitored for every 15 min in an hour.

Calculation of cell lysis rate using the following

$${\text{Cell lysis rate}}\,( {{\rm min}^{-1}}) = {{\left[ {{\text{Ln}}\left( {\text{ODt}} \right) - {\text{Ln}}\left( {{\text{OD}}_{ 0} } \right)} \right]} {\Delta \,{\text{time}}}}$$

Ln (ODt) is the natural log of the OD_600_ of vial at time t; Ln (OD_0_) is the natural log of the OD_600_ of the vial at time 0; Δ time is the total time of incubation. The plotted logarithmic graph of the OD_600_ values gives the slope, which is considered the cell lysis rate.

### Other analytical methods

As for OD_600_, samples were taken at regular intervals and were measured using a Beckman coulter model DU720 spectrophotometer. Broth samples were diluted appropriately with dH_2_O to give a raw OD_600_ value between 0.1 and 0.4 and were corrected with the dilution factor.

The *S. cerevisiae* cell viability was measured using a BD FACS Calibur Flow Cytometer (BD Bioscience, San Jose, CA, US) with some modifications [1]. Samples taken from the culture were immediately diluted with phosphate buffered saline (PBS; pH 7.2) to a final concentration of ~10^6^ cells mL^−1^ and stained with the red fluorescence dye, propidium iodide (PI). This fluorophore only stains the DNA of nonviable cells. The cell viability was reported as the inverse of the percentage of PI-positive cells with respect to the total number of cells.

## Metabolite profiling

### Study design

Samples were obtained from both 10 L and 10,000 L bioreactors. A total of five media replicates (or spent media) for each scale were collected at EFT 46, 54, 64, 76 and 80 h, and frozen at −80 °C, and later analyzed for metabolite profiling at Metabolon, Inc. (Durham, NC, US). Additionally, one batch medium sample and one glucose feed sample were included for reference. A total of 52 samples were analyzed.

### Sample preparation

Spent medium samples were thawed on ice, and a 100 μL volume sample was used for extraction. Samples were prepared for the appropriate instrument, either liquid chromatography/mass spectrometry/mass spectrometry (LC/MS/MS) or gas chromatography/mass spectrometry (GC/MS) as described in Fu et al. [1].

### Liquid chromatography/mass spectrometry and gas chromatography/mass spectrometry

The LC/MS portion of the platform incorporated a Waters Acquity UHPLC system and a Thermo-Finnigan LTQ mass spectrometer, including an electrospray ionization source and linear ion-trap mass analyzer were used to analyze all the samples. Aliquots of samples which were vacuum-dried, reconstituted; and run through the different analytical chromatographic columns are detailed in Fu et al. [1].

### Compound identification, quantification, and data curation

Metabolites were identified by automated comparison of the ion features in the experimental samples to a reference library of over 3000 purified, authenticated chemical standard entries that include retention time, molecular weight (m/z), preferred adducts, and in-source fragments as well as their associated MS/MS^2^ spectra. In brief, samples were extracted and split into equal parts for analysis on the GC/MS and two LC/MS/MS platforms. Proprietary software was used to match ions to the in-house library of standards for metabolite identification and for metabolite quantization by peak area integration.

### Data collection, normalization and visualization

A client matrix composed of small aliquots of all sample extracts in this study was created. The client matrix technical replicate samples were treated independently throughout the process. All process samples (client matrix, and a mixture of organic components used to assess GC column performance, process blanks, etc.) were spaced evenly among the injections for each day and all samples were randomly distributed throughout each day’s run. Data were collected over multiple platform run days and thus, ‘block normalized’ by calculating the median values for each run-day block for each individual compound. This minimizes any inter-day instrument gain or drift, but does not interfere with intra-day sample variability. Missing values (if any) were assumed to be below the level of detection for that biochemical with the instrumentation used and were imputed with the observed minimum for that particular biochemical.

For visualization of biochemical differences between the various treatment groups, the data are displayed in line plot graph format and scaled such that the median value measured across all samples set to 1.0. The data selected for display by line plot were filtered by statistics or included for completion of a biochemical pathway.

### Data statistical analyses

Biochemical data were analyzed by Welch’s two-sample *t*-tests to test that the means of two independent groups are equal. The heat map tables are color coded, and descriptions are shown in Additional file 1: Figure S1. Often Welch’s correction was applied to allow for unequal variances between the groups. The *p* value relates the probability of obtaining a result as or more extreme than the observed data; a low *p* value (*p* ≤ 0.05) is generally accepted as strong evidence that the two means are different. The *q* value describes the false discovery rate; a low *q* value (*q* ≤ 0.10) is an indication of high confidence in a result.

In the case of the pathway heat map table, this format is associated with the statistical analysis of the data. Statistical cut-offs are typically employed for determining candidate metabolites that might be physiologically significant in a given study. The relatively conservative criteria of *p* ≤ 0.05 and *q* ≤ 0.10 are routinely used in metabolomics studies, which allows for the identification of metabolites that are significantly altered in response to a treatment or significantly different between two comparison groups and would be expected to yield a false discovery rate of no more than 10 %. For the purposes of this study, comparisons between the various treatment groups were taken as significant when *p* ≤ 0.05, regardless of the *q* value. This approach was taken in order to be more inclusive of data that otherwise might not meet the strict statistical cut-off values.

## Additional file

10.1186/s12934-016-0542-3 Visualization of the metabolite profiling data in line plot graph and the heat map table. Data are scaled such that the median value measured across all samples was set to 1.0. A) Line plot graph. The data for each bioreactor scale is designated as shown (10 L = green line, 10,000 L = yellow line, basal media “M” = blue point, feed media “F” = grey point). B) Heat map table analysis.

## Abbreviations

DCS: distributed control system
DO: dissolved oxygen
OD_600_: optical density at 600 nm
EtOH: ethanol
ER: endoplasmic reticulum
IPP: isopentenyl-pyrophosphate
DolP: dolichol-phosphate
GlcNAc: *N*-acetyl-glucosamine
GalNAc: *N*-acetyl-galactosamine
HMG-CoA: 3-hydroxy-3-methly-glutaryl-CoA
CoA: coenzyme A
PGI: glycosyl phosphatidyl inositol
Pir-CWPs: proteins with internal repeats within the family of cell wall proteins
G6P: glucose-6-phosphate
F6P: fructose-6-phosphate
Man6P: mannose-6-phosphate
UDP: uridine-diphosphate
GDP: guanidine-diphosphate
ATP: adenosine-triphosphate
NAD^+^: nicotinamide adenosine dinucleotide
FAD^+^: flavin adenine dinucleotide
NADH/FADH: reduced forms of NAD^+^/FAD^+^
DHAP: dihydroxyacetone-phosphate
Fruc-1, 6-biP: fructose-1,6-biphosphate
*k*_L_a: mass transfer coefficient
LIT: linear ion-trap
OTR: oxygen transfer rate
RQ: respiratory quotient
slpm: standard liter per minute
TCA: tricarboxylic acid
CDW: cell dry weight
WCW: wet cell weight
L: liter
µ: specific growth rate
EFT: elapsed fermentation time
PRD: production
LN or Ln: natural logarithm
