# Supplementary material for: Hypoxia-elicited impairment of cell wall integrity, glycosylation precursor synthesis, and growth in scaled-up high-cell density fed-batch cultures of Saccharomyces cerevisiae
Source: Microb Cell Fact. 2016 Aug 15;15:142. doi: 10.1186/s12934-016-0542-3 (PMC4986208; doi:10.1186/s12934-016-0542-3)
Supplement: Supplementary file 1 — 10.1186/s12934-016-0542-3 Visualization of the metabolite profiling data in line plot graph and the heat map table. Data are scaled such that the median value measured across all samples was set to 1.0. A) Line plot graph. The data for each bioreactor scale is designated as shown (10 L = green line, 10,000 L = yellow line, basal media “M” = blue point, feed media “F” = grey point). B) Heat map table analysis. [file 12934_2016_542_MOESM1_ESM.docx]

**Figure S1**

A)

B)
